# Comprehensive assessment of paddy soil quality under land consolidation: a novel perspective of microbiology

**DOI:** 10.7717/peerj.7351

**Published:** 2019-07-22

**Authors:** Yaoben Lin, Yanmei Ye, Cifang Wu, Jianhui Yang, Yiming Hu, Haokun Shi

**Affiliations:** 1 Land Academy for National Development, Zhejiang University, Hangzhou, Zhejiang, China; 2 Land Ecological Restoration Engineering Technology Research Center of Shandong Province, Binzhou, Shandong, China; 3 China Institute of Regulation Research, Zhejiang University of Finance & Economics, Hangzhou, Zhejiang, China

**Keywords:** Land consolidation, Soil quality, Comprehensive assessment, Microbiology

## Abstract

Soil quality assessment is an important means to demonstrate how effective land consolidation is. However, the existing assessment system is not sufficient to reflect actual soil quality. So, the purpose of this study is to integrate abiological and biological indicators into a comprehensive assessment to evaluate the paddy soil quality under different land consolidation practices. Soil samples were collected from 35 paddy sites under different land consolidation practices including land merging, land leveling (LL), ditch construction (DC) and application of organic fertilizer (AO). A total of 10 paddy sites were selected under conventional tillage (CT) from non-land consolidation area as a control group in Y county, China. The results indicated that soil organic matter (OM), total nitrogen (TN), available phosphorus, bacterial functional diversity (BFD), bacterial and fungal abundances were significantly improved. Fields under LL, among all the land consolidation practices, might still face the risk of land degradation caused by low TN, OM and microbial diversity. High microbial biomass, BFD and OM were significantly higher in fields under AO in nutrient cycle. According to the results of comprehensive assessment, the samples with severe heavy metal contamination and low microbial diversity were generally concentrated in CT. These results indicated that land consolidation was an efficient technique to improve soil quality and could achieve higher quality of agricultural products.

## Introduction

China has a history of paddy cultivation for more than 8,000 years. Paddy fields are the most important source of grain, and help maintain soil function. Thus, the quality of paddy soil is highly related to human health and ecological environment. In recent decades, heavy metal contamination, nutrient loss and microbial imbalance in paddy fields had a significant negative impact on food security (Deng et al., 2019). Therefore, the problem in paddy soil concerned the society, and the government has introduced powerful measures, including land consolidation, to govern the soil environment (Wojcik-Len et al., 2018).

Land consolidation is an effective land management method, in addition to merging fragmented land, improving agricultural facilities and soil quality, to improve agricultural production and ecological environment. It has been widely applied in most countries of the world (Demetriou, 2016; Djanibekov & Finger, 2018; Stanczuk-Galwiaczek et al., 2018). Nowadays, in response to the increasing challenges posed by paddy fields, researchers have been focusing on multifunctional potentials of land consolidation as a means to address soil quality issues, such as soil contamination, soil erosion, nutrient loss and microbial imbalance. However, land consolidation will also affect the soil physicochemical properties, microbial communities and other soil quality indicators, especially soil microbial diversity (Legrand et al., 2018; Muchova, Konc & Petrovic, 2018).

As a very precious non-renewable resource, soil plays an important role in food safety and ecological environment protection (Li, Zhang & Liu, 2019; Martin, Uroz & Barker, 2017; De Paul Obade, 2019). Soil quality definition includes the physical, chemical and biological soil entities. Abiotic and biotic interaction, however, would easily be affected by land consolidation (Wang et al., 2018b; Zeng et al., 2018). Soil microbial communities play a critical role in organic matter (OM) decomposition and nutrient cycling of carbon, nitrogen and phosphorus. They could respond quickly to the environmental changes (Bender, Wagg & Van Der Heijden, 2016; Nottingham et al., 2018; Yu et al., 2018). The diversity and composition of the soil microbial community is directly related to its function, structure and aggregation (Bender, Wagg & Van Der Heijden, 2016; Guo et al., 2019). Meanwhile, the soil environmental variables such as pH, organic carbon and heavy metals are extremely influential on determining soil microbial composition and diversity (Jiang et al., 2019; Menendez-Serra et al., 2019). However, soil quality assessment through individual physical, chemical or microbiological properties is still limited, as many properties are interrelated and the results are thus unreliable (Greiner et al., 2017; Mukhopadhyay, Maiti & Masto, 2014). In order to assess the mine soil quality, indicators such as microbial indices and soil physicochemical property indices have been integrated into the soil quality index system (Li et al., 2018b). Some researchers have attempted to assess the soil quality level of wetlands based on heavy metal contamination and fertility assessment (Wang et al., 2018a). At present, biological properties have not been included in the evaluation of paddy soil quality (PSQ) yet, which is the main reason for the unreliable results of soil quality evaluation. Although these soil quality indexes are not comprehensive (Li et al., 2018b), they are a good reference for the impact of paddy fields after land consolidation.

The purpose of this study was to comprehensively evaluate the real soil quality of paddy fields under different land consolidation practices by combining soil physical and chemical properties, heavy metal pollution and soil biological properties. In this study, the paddy soils were selected from the land consolidation area of Y county, China. The selected sites had been treated with different land consolidation practices. The results of physicochemical characteristics, heavy metal contamination and microbial indexes were compared with the soils under conventional tillage (CT) from non-land consolidation area. This research attempted to comprehensively evaluate the soil quality in the land consolidation area from the perspective of microbiology, in order to solve the existing problems in soil quality evaluation, investigate the influence of different land consolidation practices on soil quality and provide reference for soil quality protection and pollution remediation.

## Materials and Methods

### Study site and soil sampling

The study area is in Y county, in the eastern coastal area of China. It has a history of thousands of years in rice cultivation. With its excellent geographical location and climate conditions, Y county is a leading county of food production in China. However, agricultural land fragmentation and soil degradation are still a serious problem in sustainable agriculture in Y county. Therefore, in recent years, it is an important task to concentrate fragmented paddy fields and improve soil quality in this area via land consolidation. In areas under land consolidation, measures such as land merging (LM), land leveling (LL), ditch construction (DC) and application of organic fertilizer (AO) are mainly adopted to improve the soil quality.

In September 2018, soil samples were collected from 45 sites from Y county, with 35 inside the land consolidation area (over 5 years after implementing different land consolidation practices) and 10 outside but near the land consolidation area under CT (over 10 years for farming). All the sampling sites were paddy fields and all the paddies from the sampling sites were xiushui 134. The soil samples were collected from the bulk topsoil (0–10 cm) along a 100 × 100 m square (Legrand et al., 2018; Yu et al., 2018). Samples were removed of roots, stones, plant residues, animals and other things manually, then stored with 20 g per sample below −80 °C before DNA extraction, and transported 500 g per sample to the laboratory for analysis on physicochemical, heavy metals and other environmental variables (Lüneberg et al., 2018). After soil homogenization, three repeated DNA extractions and soil environmental variable analysis were performed for each soil sampling site.

### Measurements and analyses

#### Soil physical and chemical indicators

The soil pH was measured at 1:2.5 (soil:water) by pH meter and the soil water content (SWC) was determined by oven drying at 105 °C for 6 h (Baldoncini et al., 2019; De Paul Obade, 2019). The concentrations of Cu, Zn, Cd, Pb, Ni, Cr, Hg and As in soils were tested by an inductively coupled plasma source mass spectrometer (Agilent 7800; Agilent Technologies, Palo Alto, CA, USA) after being extracted (Wu et al., 2019b). The soil OM, total nitrogen (TN), available phosphorus (AP) and available kalium (AK) were measured by total organic carbon analyzer (BOCS301; Shimazu Enterprise Management (Kyoto) co., LTD, Shimadzu, Japan), automatic kieldahl apparatus (K9860; Shandong Haineng Scientific Instrument co. LTD, Jinan, China), spectrophotometer and flame photometer (Song et al., 2018), respectively.

#### Microbial diversity

The soil genomic DNA for the PCR amplification was extracted from 0.5 g of triplicate soil samples using the FastDNA SPIN kit (MP Biomedicals, Irvine, CA, USA) according to the manufacturer’s instructions. The quality and concentration of extracted DNA were determined by using the Nanodrop 2000 spectrophotometer (Thermo Scientific, Waltham, MA, USA), and the DNA was stored at −20 °C for further use (Ma et al., 2018). The V3–V4 hypervariable regions of bacterial 16SrRNA gene were amplified with the primer pairs 338F and 806R, and the Internal Transcribed Spacer (ITS) regions of the fungal DNA gene were amplified using the primer pairs ITS1F and IT2R (Caban et al., 2018; Nottingham et al., 2018). PCR of bacteria and fungi were all performed to amplify 10 ng of template DNA in a 20 μl reaction system containing 0.8 μl (five μM) of each primer. The PCR reactions were performed by ABI GeneAmp® 9700 with the following program: 95 °C for 3 min; 10 cycles at 95 °C for 30 s, 60 °C for 30 s and 72 °C for 45 s, then a final extension at 72 °C for 10 min and 10 °C for 30 min. Sequencing data were performed on Illumina HiSeq4000 and the raw DNA sequencing data were processed with Quantitative Insights Into Microbial Ecology 2 (QIIME 2) (Jiang et al., 2019; Nottingham et al., 2018). Sequences were clustered into operational taxonomic units (OTUs) at 97% similarity, and the most abundant sequence was selected from each OTU to represent the respective OTU. Finally, the representative sequences were deposited into the NCBI SRA database (accession number: PRJNA532482, PRJNA532470).

The indexes of microbial alpha-diversity were estimated by mothur package (version v.1.30.1), including Shannon and Chao 1 (Barnes et al., 2017; Shao et al., 2019). The student’s test was used to estimate significant differences in diversity indexes between the different groups. And the beta-diversity analyses, including hierarchical clustering, principal component analysis, principal co-ordinates analysis and redundancy analysis, were calculated by QIIME 2 and software R (version 2.1.3) (Jiang et al., 2019).

Furthermore, some bacteria, among the beneficial microbes in the soil, can produce indole acetic acid to increase chlorophyll content, and as a result, increase the photosynthesis rate in the plant. Some other microbes can generate biologically active substances, such as hormones and enzymes, to control soil diseases and accelerate the circulation of nutrients in the soil, thereby improving the crop growth (Antoun, 2012). Therefore, the functions of the beneficial microorganisms play a crucial role in assessing soil quality in the paddy fields. The bacteria such as *Azospirillum*, *Bacillus*, *Bradyrhizobium*, *Escherichia-Shigella*, *Mesorhizobium*, *Pseudomonas*, *Rhizobium*, *Rhodopseudomonas* and *Streptomyces*, as well as fungi such as *Glomerales*, *Mucorales*, *Orbiliales*, *Tremellales* and *Hypocreales* are the beneficial microbes in the soil according to the previous researches (Antoun, 2012; Chen et al., 2012; Hayat et al., 2010).

#### Functional diversity of the bacterial community

16S rRNA gene is a key tool for studying the functional capabilities of microbial communities (Langille et al., 2013). It is necessary to figure out how the information about the soil bacteria helps us predict the extent of effects that soil bacteria may have on farmland ecosystems (Fierer, 2017). The 16S function prediction is a method to standardize the OTU abundance table by the Phylogenetic Investigation of Communities by Reconstruction of Unobserved States (PICRUSt) software which stores the COG information and KEGG Ortholog (KO) information related to the greengene id. Then, the COG family information and KO information relevant to the OUT were obtained by the greengene id related to each OUT, and the abundance and KO abundance of each COG was calculated. According to the information of the COG database, the description and function of each COG can be analyzed from the eggNOG database, thereby obtaining a functional abundance spectrum. The information of KO, Pathway and EC can be obtained from the KEGG database, then the abundance of each functional category can be calculated according to the OTU abundance. In addition, for Pathway, PICRUSt can be used to obtain three levels of information on metabolic pathways and obtain abundance tables for each level (Malik et al., 2018).

#### Microbial biomass

The soil microbial biomass carbon (MBC), microbial biomass nitrogen (MBN) and microbial biomass phosphorus (MBP) were measured by chloroform fumigation extraction based on moist paddy soil put forward by Vance (Li et al., 2018a). Briefly, two moist soil samples (five g dry weight equivalent) were inoculated in 150 ml centrifuge tubes. Then, one sample was fumigated with ethanol-free CHCl_3_ for 24 h. Both fumigated and non-fumigated soils were extracted with a 20 ml 0.5M K_2_SO_4_ solution by 30 min horizontal shaking and filtering. The organic carbon and TN in the extracts were determined by a C/N automatic analyzer for catalytic high temperature combustion. Then MBC, MBN and MBP were determined by using conversion factors of 0.45, 0.54 and 0.4, respectively (Murugan et al., 2019).

### Statistical analysis

Index system for PSQ could be established by minimum data set (MDS), fuzzy logic model and the geoaccumulation index (GI; Wang et al., 2018a; Wu et al., 2019a).

#### Minimum data set

The MDS was considered to be a reliable and measurable set of soil assessment indicators that eliminate redundancy and reduce information loss (Wang et al., 2018a).

First, statistical characteristics, variabilities of each indicator and correlations between different indicators are evaluated, then the results are used as a reference to select indicators. Second, the indicators are grouped by PCA in case of data redundancy, and only components with eigenvalues ≥1 are selected. Third, the vector norm value for each indicator is calculated to filter the indicators. Because the vector norm value represents the combined loading of one indicator in all components. The formula is as follows:

(1) $${\rm{}}{N_{{\rm{\alpha \beta }}}} = \sqrt {\mathop \sum \limits_{{\rm{\alpha }} = 1}^{\rm{\beta }} \left( {{{\rm{\lambda }}_{\rm{\beta }}}{\rm{\mu }}_{{\rm{\alpha \beta }}}^2} \right)} $$

μ_αβ_, λ_β_ are the loading value and eigenvalue in component β, respectively.

Finally, normal linear transformation is conducted on the norm value and correlation of each indicator to calculate the sum. Indicators whose values are greater than 90% of the maximum are selected for MDS.

#### Calculation method

##### Fuzzy logic model

Most of the indicators in MDS could be scored by linear scoring functions of fuzzy logic model to eliminate the interference of different indicator units (Joss et al., 2008). Two scoring equations were used to convert the indicators to certain values between 0 and 1. And the threshold values of each indicator were determined by referring to the previous researches (De Paul Obade & Lal, 2016). The scoring equations are as follows:

(2) $$f\left( x \right) = \left\{ {\matrix{
1 & , & {x \ge {b_1}} \cr
{{{x - {a_1}} \over {{b_1} - {a_1}}}\,} & , & {{a_1} < x < {b_1}} \cr
0 & , & {x \le {a_1}} \cr
} } \right.$$

(3) $$f\left( x \right) = \left\{ {\matrix{
1 & , & {{b_2} \ge x \ge {a_2}} \cr
{{{x - {a_1}} \over {{a_2} - {a_1}}}} & , & {{a_1} < x < {a_2}} \cr
{{{x - {b_1}} \over {{b_2} - {b_1}}}} & , & {{b_1} > x > {b_2}} \cr
{0\,} & , & {x \le {a_1}\,{\rm{or}}\,x \ge {b_1}} \cr
} } \right.$$

According to the formulas, *x* is the measuring value of indicators and *f*(*x*) is the corresponding score of the indicators ranging between 0 and 1. In addition, *a*_1_ and *b*_1_ are the lower and upper threshold values of indicators; *a*_2_ and *b*_2_ are the lower and upper limits of the optimum values of indicators. In addition, the Eq. (2) is applicable to evaluate the scores of indicators like SWC, OM, TN, AK, AP, Shannon of bacteria (SB), Chao 1 of bacteria (CB), Shannon of fungus (SF), Chao 1 of fungus and bacterial functional diversity (BFD); and the Eq. (3) is suitable to assess the scores of pH, slit, clay, sand, MBP, MBC and MBN (Liu et al., 2014; Wang et al., 2018a).

##### The geoaccumulation index

Globally, the GI is widely used to assess the levels of heavy metal contamination in soils as a standard compared with pre-industrial levels. The formula is as follows:

(4) $${\rm{G}}{{\rm{I}}_i} = {\rm{lo}}{{\rm{g}}_2}\left({{{{M_i}} \over {1.5{N_i}}}} \right)$$

*M_i_* is the concentration of a single heavy metal in the soil from sample *i*; *N_i_* is geochemical background value of single heavy metal in soil samples in this area (Fan & Wang, 2009); and the extent of environmental and human activities that affect the fluctuations of metal contents is reflected by the coefficient (1.5) (Keshavarzi & Kumar, 2018).

Then a computational model can be established to obtain a reasonable and comprehensive pollution assessment (PA) according to the results of GI. The formula is as follows:

(5) $${\rm{PA}} = \sqrt {{{{\rm{G}}{{\rm{I}}_i}_{{\rm{ave}}}^2 + {\rm{G}}{{\rm{I}}_i}_{{\rm{max}}}^2} \over 2}} $$

GI_*i*ave_ and GI_*i*max_ represent the average and maximum values of GI_*i*_ for the eight heavy metals, respectively.

#### Comprehensive assessment of paddy soil quality

Multi-standard quantitation procedures were applied to the comprehensive assessment of PSQ, including soil physicochemical properties, biological indicators, and heavy metal contamination. The indicators of soil physicochemical and biological properties can be integrated into the Eq. (6) based on the results of *f*(*x*) in Eqs. (2) and (3). The formula is as follows:

(6) $${\rm{PB}} = \mathop \sum \limits_{i = 1}^n f{\left( x \right)_i} \times {W_i}$$

PB is the integrated results of soil physicochemical and biological properties; and *f*(*x*)_*i*_ and *W_i_* are the score and weight of the indicator *i* in MDS, respectively. In this study, the weights of the indicators were determined by the common factors from PCA.

Meanwhile, the levels of heavy metal contamination should be changed from Eq. (4) as follows:

(7) $${\rm{HM}} = \left\{ {\matrix{
{1\quad\quad\quad\quad\quad,} \hfill & {\left( {{\rm{G}}{{\rm{I}}_i} < 0} \right)} \hfill \cr
{1/\left( {{\rm{G}}{{\rm{I}}_i} + 1} \right),} \hfill & {\left( {{\rm{G}}{{\rm{I}}_i} \ge 0} \right)} \hfill \cr
} } \right.$$

HM represents the levels of the standardized environment index, while GI_*i*_ is the GI from Eq. (4).

Therefore, the PSQ is illustrated by the results mentioned above, and the formula is as follows:

(8) $${\rm{PSQ}} = {\rm{MIN}}\left\{ {\sqrt {{{\left({{\rm{PB}}_{{\rm{ave}}}^2 + {\rm{PB}}_{{\rm{min}}}^2} \right)} \over 2}}, \sqrt {{{\left({{\rm{HM}}_{{\rm{ave}}}^2 + {\rm{HM}}_{{\rm{min}}}^2} \right)} \over 2}} } \right\}$$

PB_ave_ represents the average PB value and PB_min_ represents the minimum BP values; while HM_ave_ is the average HM value and HM_min_ is the minimum HM values.

## Results

### Soil physical and chemical indicators

#### Soil physiochemical properties

There were 35 fields under land consolidation (LM, LL, DC and AO), and 10 other fields under CT from non-land consolidation area. All the 45 fields had similar paddy management practices and the soils had analogous textures too. The soil physiochemical properties of the samples from the study area are listed in Table 1.

**Table 1:** The physiochemical properties of soils under different land consolidation practices.

Indicator	LM	LL	DC	AO	CT	Max	Min	Mean	CV (%)
pH	7.15 ± 0.53	7.28 ± 0.57	7.18 ± 0.57	7.22 ± 0.61	7.38 ± 0.71	8.32	6.17	7.3	0.08
OM (g/kg)	42.4 ± 15.8	41.1 ± 16	48.2 ± 14.4	53.3 ± 10.1	30.2 ± 9.8	70.4	16	39.9	0.37
SWC (%)	39.6 ± 8.1	39.7 ± 8.6	44.8 ± 5.5	41.5 ± 7.1	30.3 ± 5.7	54.2	20.7	37.6	0.22
TN (g/kg)	2.3 ± 0.8	2.2 ± 0.8	2.5 ± 0.7	2.7 ± 0.6	1.7 ± 0.5	3.75	0.93	2.11	0.34
AP (mg/kg)	70.2 ± 58.1	70.6 ± 54.6	86.7 ± 82.8	95.9 ± 88.8	53.4 ± 37.1	330.8	8.56	70.69	0.94
AK (μg/ml)	28.8 ± 16.1	29.6 ± 12.2	30.2 ± 14.3	33.5 ± 15.9	34 ± 14.9	80.25	14.45	30.49	0.45
Silt (%)	34.3 ± 10.5	30.1 ± 4.5	39.3 ± 7.2	35.2 ± 9.2	28.3 ± 5.3	54.21	19.16	36.91	0.2
Clay (%)	35.6 ± 6.7	34.2 ± 13.3	27.2 ± 8.2	30.3 ± 7.4	40.7 ± 0.69	43.13	22.34	28.77	0.14
Sand (%)	35.3 ± 7.2	34.1 ± 10.2	28.3 ± 6.7	32.5 ± 7.2	38.2 ± 10.2	53.78	7.2	32.61	0.53

**Notes:**

The values are reported as mean values ± standard deviation.

OM, organic matter; SWC, soil water content; TN, total nitrogen; AP, available phosphorus; AK, available kalium; LM, land merging; LL, land leveling; DC, ditch construction; AO, application of organic fertilizer; CT, conventional tillage; VC, coefficient of variation.

#### Soil heavy metals

The heavy metal concentrations of the paddy soils and the reference background values of this area are listed in Table 2. On average, the Zn was the main metal, accounting for about 42%, and was ranked by concentrations as follows: Zn > Cr > As > Ni > Cu > Pb > Cd. The concentrations of Cu, Zn, Cd, Ni and As were higher than the background values at all sites, while the concentrations of Pb were lower than the background values at some sites under land consolidation practices of LM and LL. The concentrations of Cr in soils from fields under land consolidation practices of DC and AO were much lower than other fields. The results also suggested the paddy soils in the fields under land consolidation were less polluted by the heavy metals than fields under CT.

**Table 2:** Heavy metal concentrations in paddy soils (mg/kg).

	Cu	Cd	Pb	Cr	As	Hg	Ni	Zn
LM	41.72 ± 8.83	2.27 ± 0.33	37.62 ± 12.06	212.64 ± 11.22	4.99 ± 3.91	0.53 ± 0.25	54.55 ± 7.44	112.51 ± 21.58
LL	39.44 ± 7.92	2.23 ± 0.27	37.54 ± 12.81	214.61 ± 11.34	4.21 ± 3.51	0.45 ± 0.15	54.06 ± 7.63	110.47 ± 18.39
DC	45.95 ± 12.74	2.28 ± 0.23	43.39 ± 13.23	214.65 ± 11.67	4.66 ± 3.83	0.57 ± 0.28	53.15 ± 5.60	125.75 ± 26.70
AO	46.55 ± 12.25	2.27 ± 0.31	42.98 ± 10.38	216.45 ± 11.12	3.89 ± 2.90	0.53 ± 0.21	55.00 ± 6.86	128.36 ± 24.72
CT	48.97 ± 6.71	3.30 ± 0.55	46.83 ± 8.81	252.96 ± 11.05	7.88 ± 5.29	0.65 ± 0.37	64 ± 10.85	136.82 ± 21.17
Max	84	3.98	77.5	268.8	14.4	1.38	80.4	198
Min	25	1.57	20	195	0.81	0.1	39	70.6
Mean	45.17	2.5	41.94	223.79	5.29	0.55	56.77	123.27
CV (%)	22.38	22.86	26.4	8.72	77.91	49.99	15.96	20.7
Background	22.6	0.17	35.7	56	6.9	0.17	23.9	83.1

**Notes:**

The concentrations are reported as mean values ± standard deviation.

LM, land merging; LL, land leveling; DC, ditch construction; AO, application of organic fertilizer; CT, conventional tillage; VC, coefficient of variation.

### Soil biological indicators

#### Microbial biomass

The concentrations of MBP, MBC and MBN showed an increase after land consolidation, with different land consolidation practices contributing more than 18% of value growth. The concentrations of MBP, MBC and MBN were the highest under the practice of AO, and the concentrations of MBP and MBC were significantly higher than other practices (Table 3).

**Table 3:** Soil biochemical biomass properties under land consolidation practices.

	MBP (mg/kg)	MBC (mg/kg)	MBN (mg/kg)
AO	45.15 ± 17.73^a^	856.44 ± 291.88^a^	49.66 ± 29.48^a^
DC	38.77 ± 16.47^ab^	687.01 ± 229.00^b^	39.74 ± 21.38^a^
LL	33.65 ± 14.16^b^	668.33 ± 300.65^b^	43.71 ± 24.79^a^
LM	35.42 ± 19.59^ab^	646.73 ± 258.21^b^	39.40 ± 20.48^a^
CT	20.13 ± 7.90^c^	404.00 ± 162.04^c^	23.45 ± 5.57^b^
CV (%)	48.55	41.85	57.77

**Note:**

The values are reported as mean values ± standard deviation; different letters indicate significant differences of one indicator under different land consolidation practices (LSD test, *p* < 0.05).

#### Microbial diversity

**Figure 1:**
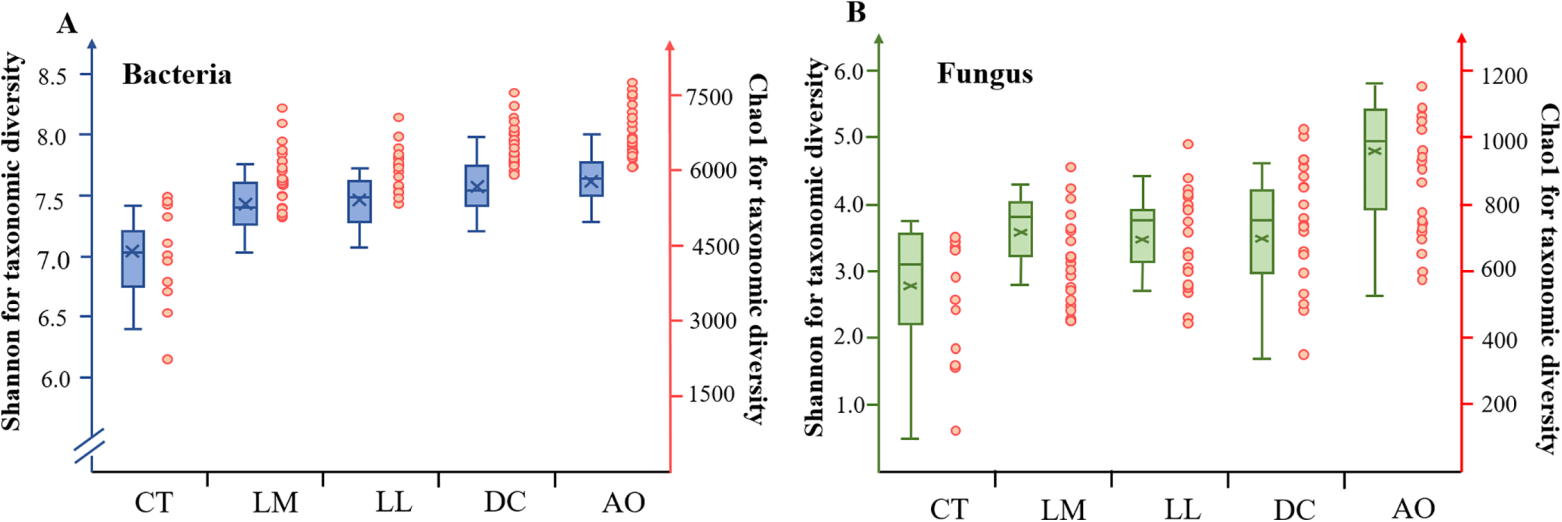
The microbial abundance and diversity for all soil samples. Box plots and dots explain the Shannon and Chao indices of bacteria (A) and fungi (B), respectively.

The student’s test revealed that the main factor which significantly affected the diversity of bacterial communities was land consolidation, including LM, LL, DC and AO (*p* < 0.01). The microbial community richness indices such as Chao 1 and the diversity indices like Shannon were significantly higher in the soils collected from land consolidation area compared with non-land consolidation areas (*p* < 0.01) (Fig. 1). Meanwhile, most of the alpha-diversity indices were also significantly higher under the AO (*p* < 0.01).

Similar to the bacterial communities, most of the alpha-diversity indices of the fungal communities were significantly higher in the soil samples from land consolidation area compared with non-land consolidation area (Student’s *t*-test, *p* < 0.01). The community richness index—Chao 1, and the community diversity index—Shannon, were significantly higher in soils from land consolidation area (*p* < 0.01). Meanwhile, the AO also significantly increased the alpha-diversity indices of the fungal communities, except for Shannon. Most of the alpha-diversity indices were also significantly higher in the large-scale field than in small-scale field.

**Figure 2:**
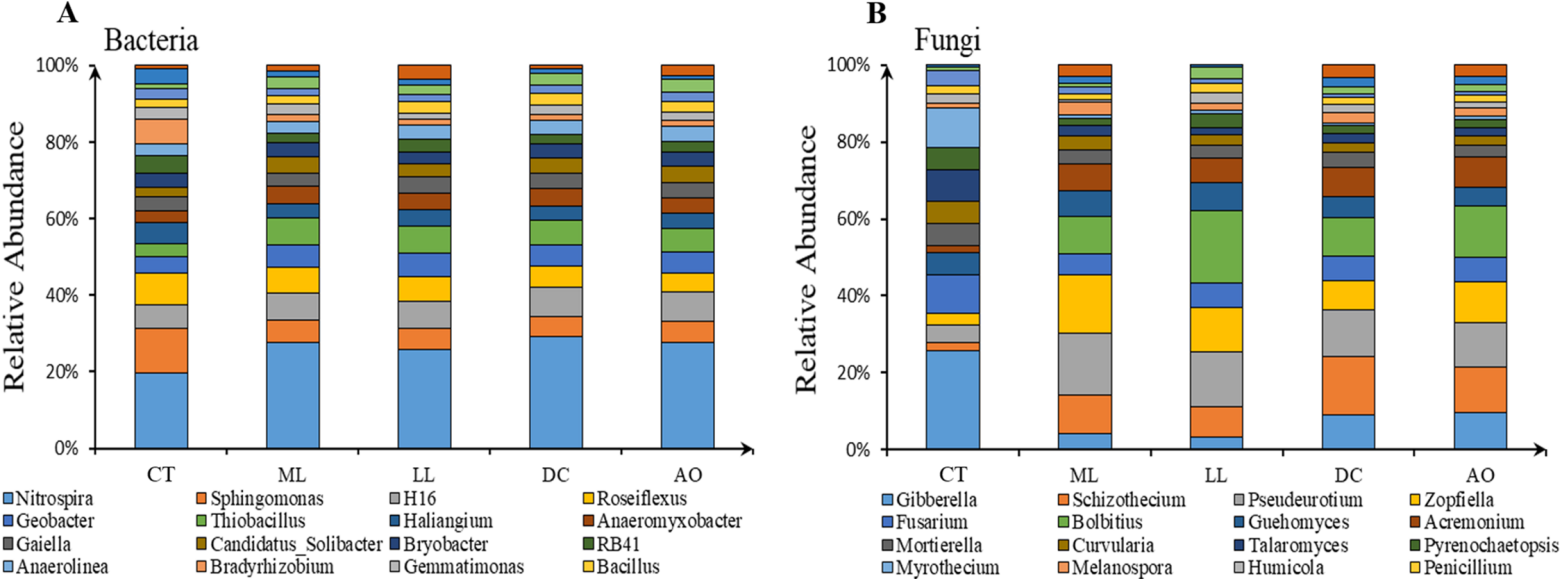
The structure of main soil bacteria (A) and fungi (B) under different land consolidation practices.

A total of 2,048,688 valid sequences of 16S rDNA were clustered into 11,244 OTUs, which were assigned to 56 phyla and 1,100 genera for bacteria. The OTUs allocated above the phylum level and genus level were approximately 99% and 35%, respectively. Proteobacteria (32.11% of OTUs), Chloroflexi (19.04% of OTUs), Acidobacteria (14.50% of OTUs), Actinobacteria (8.14% of OTUs), Bacteroidetes (5.18% of OTUs) and Nitrospirae (4.31% of OTUs) represented more than 80% of the described bacterial phyla. At genus level, Nitrospira, Sphingomonas, H16, Roseiflexus, Geobacter, Thiobacillus, Haliangium, Anaeromyxobacter, Gaiella and Candidatus_Solibacter were the most abundant genera (Fig. 2).

A total of 2,573,968 valid sequences of ITS were clustered into 5,377 OTUs and assigned to seven phyla and 457 genera for fungi. In addition to unclassified fungi, approximately 95% of the OTUs were assigned to Ascomycota (76.75%), Basidiomycota (13.17%) and Rozellomycota (5.73%). The predominant fungi genera were Gibberella (3.44% of OTUs), Schizothecium (2.92% of OTUs), Pseudeurotium (2.72% of OTUs), Zopfiella (2.65% of OTUs), Fusarium (2.05% of OTUs), Bolbitius (1.92% of OTUs), Guehomyces (1.86% of OTUs), Acremonium (1.41% of OTUs), Mortierella (1.26% of OTUs) and Curvularia (1.07% of OTUs) (Fig. 2).

**Figure 3:**
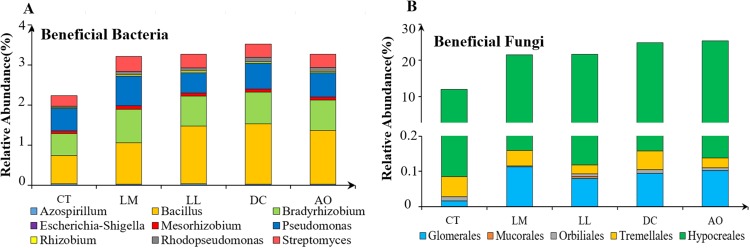
The relative abundance of beneficial bacteria (A) and fungi (B) in the soils under different land consolidation practices.

The relative abundance of beneficial microbes in the soils from land consolidation area was much higher than those from non-land consolidation area (Fig. 3).

#### Bacterial functional diversity

**Figure 4:**
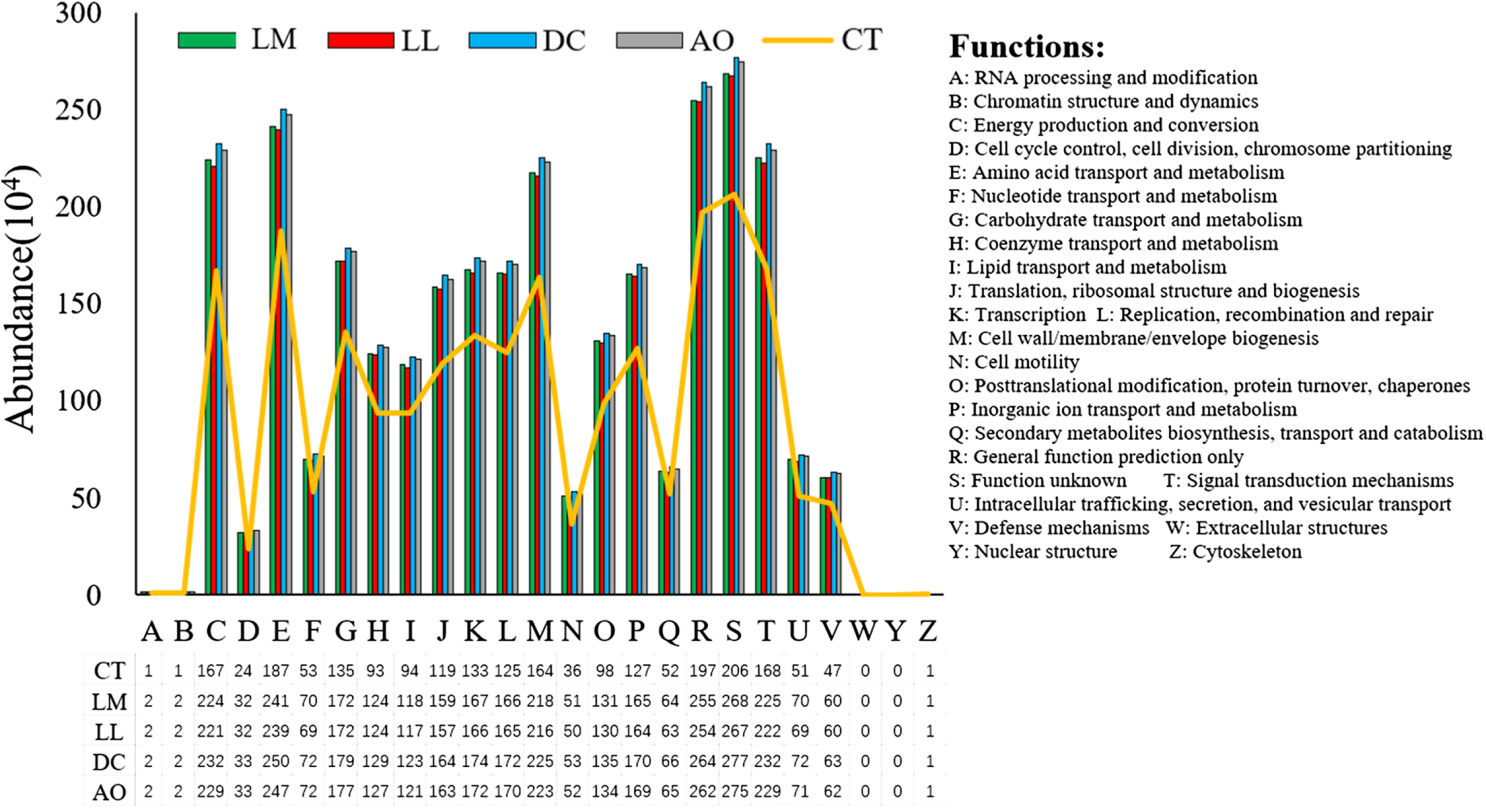
The abundance of functions inferred by PICRUSt in the soil samples under different land consolidation practices.

The 16S rRNA gene is a key tool for studying the functional capabilities of microbial communities (Langille et al., 2013). We obtained all COG and KO annotations by PICRUSt to generate a table of abundances about KO and COG for the soil bacterial genomes that have identifiers in the Greengenes reference tree (Wong et al., 2016). According to the functional profiles of soil samples, amino acid transport and metabolism, signal transduction mechanisms, energy production and conversion and cell wall/membrane/envelope biogenesis were the most abundant functions, especially in the soils under land consolidation (Fig. 4). This suggested that the functions of nitrogen cycling, denitrification and respiration in soil bacteria became more effective after land consolidation (Lopez et al., 2018). Nonetheless, the differences of the functions in the soil bacteria between the soils from land consolidation area were not significant (*p* < 0.05), which also led to the significant differences of the corresponding bacterial community compositional structures.

### Comprehensive assessment of soil quality

#### Minimum data set

**Figure 5:**
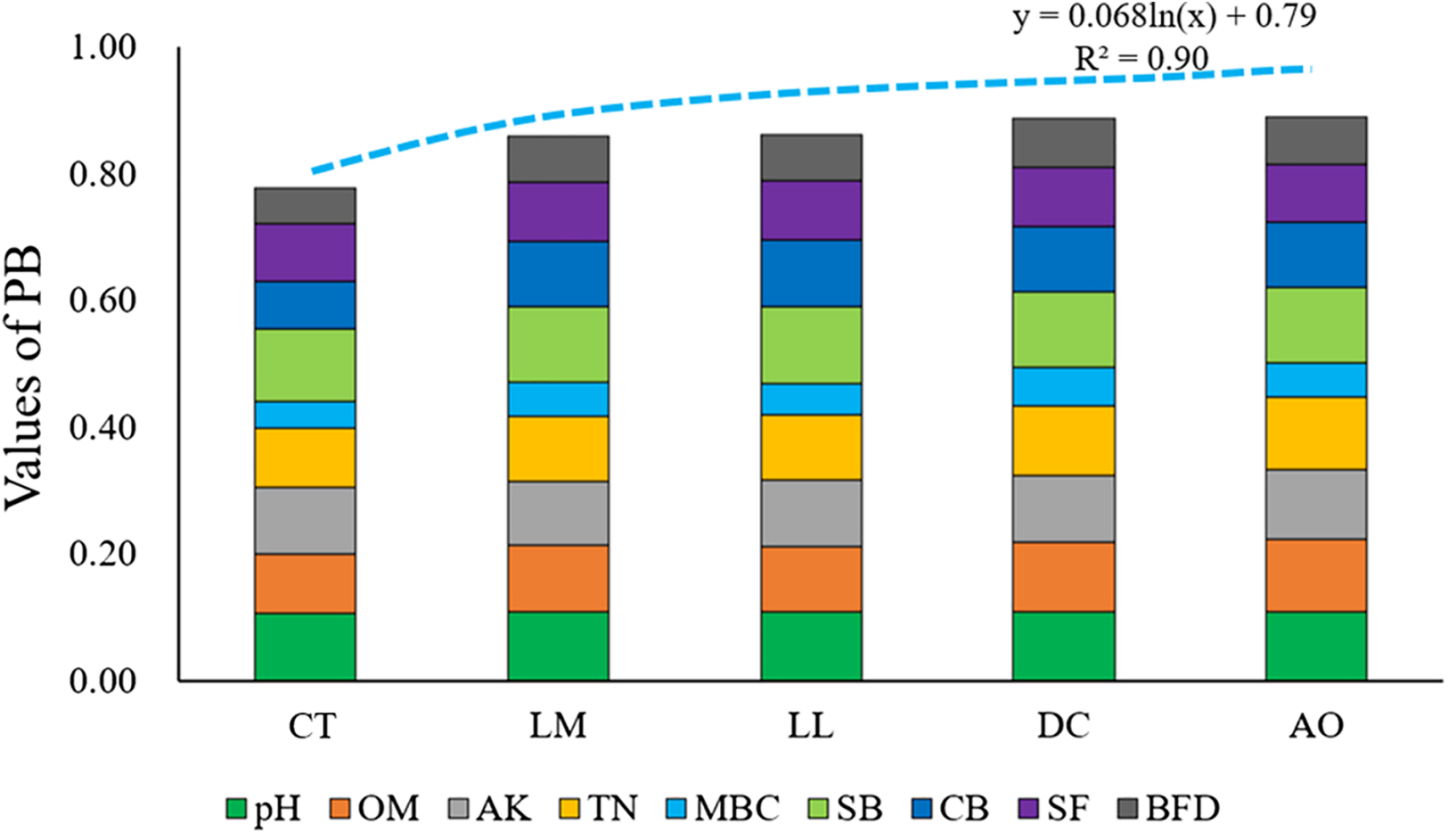
The value of PB and the individual contribution of MDS indicators under different land consolidation practices.

There were five principal components with eigenvalues >1.0 and a cumulative variance of 73.17% (Table 4). The large eigenvector values strongly correlated with each of the five primary PCs were weighted according to the percentage variance demonstrated by the particular PC. The variables with the largest absolute eigenvector values for PC1 were OM and TN. For PC2, the largest absolute eigenvector values were for AK, SB and CB. Then, MBC, BFD, SF and pH were the indicators with the largest absolute eigenvector values in the rest of principal components. The weighting values for these indicators were assigned according to the percentage variance demonstrated by the particular PC. The 25.25% from PC1 was distributed equally between OM and TN, and 18.13% from PC2 was also applied to AK, SB and CB, then the remaining percentage variances were also distributed in the same way. The weighting values were indicated by common factors through dividing each weighting value by the total weighting value of 0.7317. The final calculation of PB formula was showed in Eq. (9). The value of PB and the individual contribution of MDS indicators under different land consolidation practices were exhibited in Fig. 5.

**Table 4:** Results of principal component analysis of soil physiochemical and microbial characteristics under different land consolidation practices.

	PC1	PC2	PC3	PC4	PC5
SWC	0.715	−0.166	0.189	−0.169	0.212
pH	−0.371	−0.063	−0.038	0.398	0.708
OM	0.819	0.305	0.2	−0.111	0.062
AP	0.371	0.599	−0.438	0.212	−0.093
AK	0.075	0.773	−0.223	0.137	0.197
TN	0.842	0.321	0.169	−0.138	−0.103
Silt	−0.263	0.125	0.172	−0.023	0.146
Clay	0.048	0.149	−0.027	−0.101	−0.239
Sand	0.206	0.131	0.052	0.024	−0.1
MBP	0.001	0.263	0.217	0.512	0.11
MBC	0.31	0.054	0.685	−0.106	0.45
MBN	0.268	0.006	−0.563	0.044	0.338
SB	0.533	−0.735	−0.241	0.145	0.097
CB	0.552	−0.725	−0.231	0.169	0.014
SF	−0.046	−0.158	0.485	0.663	−0.296
CF	0.593	0.103	−0.101	0.452	−0.23
BFD	0.569	0.321	0.673	0.321	0.498
Eigenvalue	3.282	2.356	1.536	1.251	1.087
Variance (%)	25.248	18.126	11.814	9.624	8.36
Cumulative variance (%)	25.248	43.374	55.188	64.812	73.172

(9) $$\eqalign{
& {\rm{PB}} = 0.115f\left( {{\rm{OM}},{\rm{TN}}} \right) + 0.124f\left( {{\rm{AK}},{\rm{SB}},{\rm{CB}}} \right) + 0.076f\left( {{\rm{MBC}},{\rm{BFD}}} \right) + 0.132f\left( {{\rm{SF}}} \right) + 0.114f\left( {{\rm{pH}}} \right) \cr
& \cr} $$

#### Comprehensive soil quality

**Figure 6:**
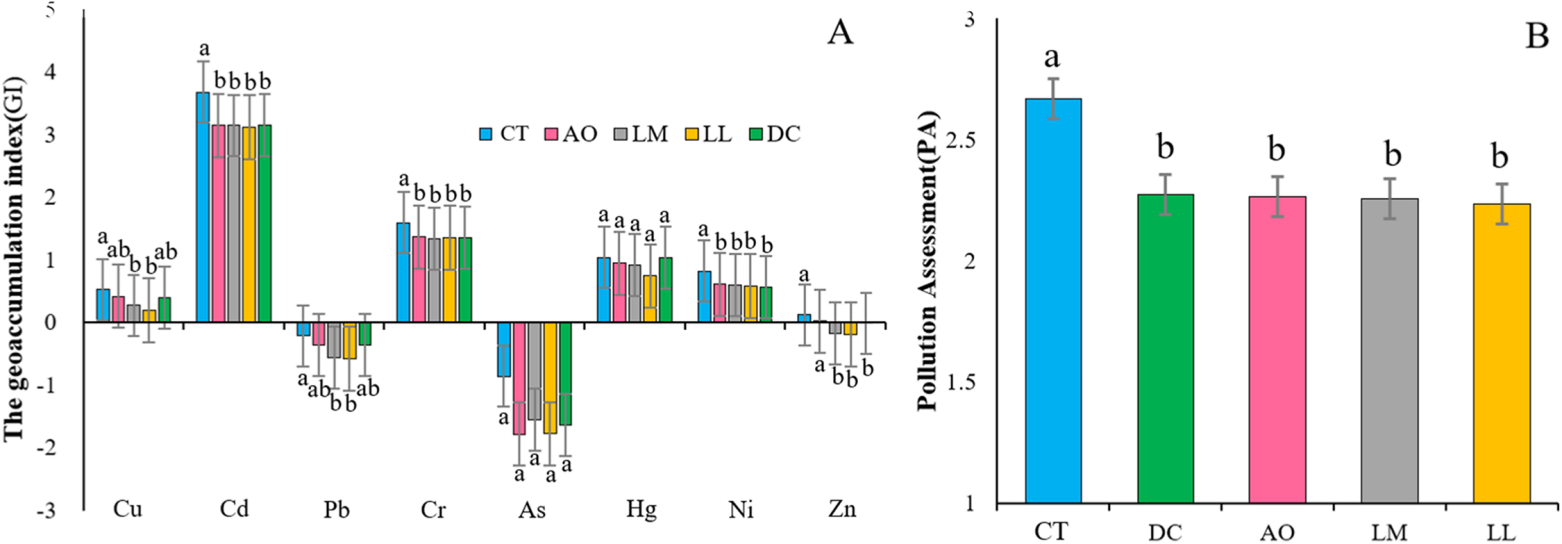
The GI values (A) and PA values (B) under different land consolidation practices. Error bars indicate the standard deviation of the sample values. Different letters on the bars indicate significant difference at *p* < 0.05.

The GI was used to assess the degree of heavy metal contamination. Among the eight heavy metals, Cd showed the highest GI values, indicating high level of pollution of Cd. In contrast, the GI values of Pb and As were both under zero, meaning that there was no contamination of Pb and As in the study area. In general, the GI values followed the order of Cd > Cr >Hg >Ni > Cu >Zn > Pb >As. At the same time, there were significant differences in the GI values of heavy metals in different land consolidation practices, except for As and Hg (Fig. 6A). All soils from non-land consolidation area (CT) have the highest GI values (Fig. 6A) and PA values (Fig. 6B), indicating that land consolidation can effectively reduce the pollution level of heavy metals.

**Figure 7:**
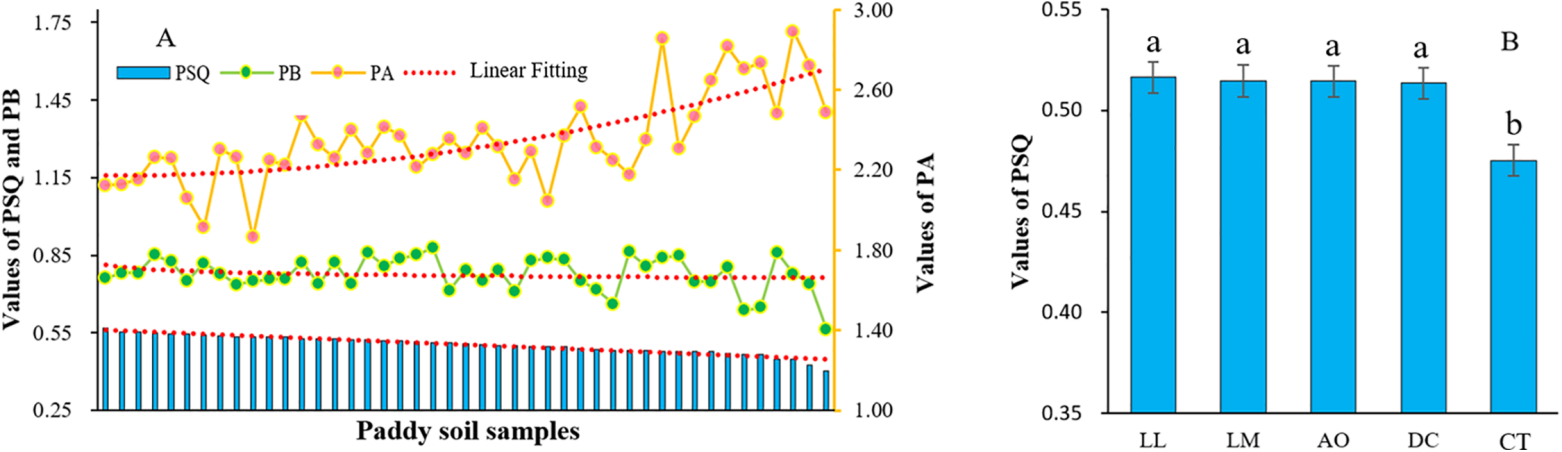
The indexes changing tendency of all paddy soil samples (A) and the PSQ values under different land consolidation practices (B).

Meanwhile, all soils samples from the study area belonged to low soil comprehensive quality level according to the results of comprehensive assessment framework (Fig. 7A). However, the PSQ values of soils under land consolidation were significantly higher than those from non-land consolidation area (Fig. 7B). The soils from CT showed the highest values of PA and the relatively low values of PB, resulting in low level of soil quality. The linear fitting in Fig. 7A was performed to test the relationship among the values of PB, PA and PSQ, and the results showed that the increased values of PB caused soil quality degradation.

## Discussion

### Soil physical and chemical indicators under different land consolidation practices

It turned out that the values of OM, SWC, TN, AP and Silt in soils under land consolidation were significantly increased compared with the soils from CT (Table 1). The AO significantly improved the soil nutrient levels of OM, TN and AP compared with other practices. This is consistent with the results of previous researches demonstrating that organic fertilizers always have a positive impact on soil quality (Kumar et al., 2017; Lazcano et al., 2013). At the same time, the soil pH in the land consolidation area was significantly decreased to approximately 7.0 because constructing ditches, the AOs and the corresponding soil management all helped to regulate the soil water cycle and pH (Ding et al., 2019).

Most soil heavy metal concentrations showed significant differences between paddy fields except for As and Hg. To be specific, the average concentrations of Cu, Cd, Pb, Cr, As, Hg, Ni and Zn were 1.99, 14.71, 1.17, 3.7, 3.99, 0.77, 3.23, 2.38 and 1.48 times greater than the background values, respectively, which exhibited severe and heterogeneous levels of anthropogenic contamination. However, land consolidation reduced about 15% heavy metal contamination according to the values of PA, indicating that land consolidation can effectively reduce the contamination level of these heavy metals, which was consistent with previous research results (Liu et al., 2015; Wang & Cheng, 2012). Because land consolidation can effectively transfer some heavy metals through promoting the water cycle by the LL and the construction of ditches, and effectively eliminate the pollution sources by means of unified management and monitoring. In addition, the AOs provides nutrients for heavy metal tolerant microorganisms to strengthen their function of absorbing and transferring heavy metals. All these land consolidation practices have effectively reduced the pollution level of heavy metals.

**Figure 8:**
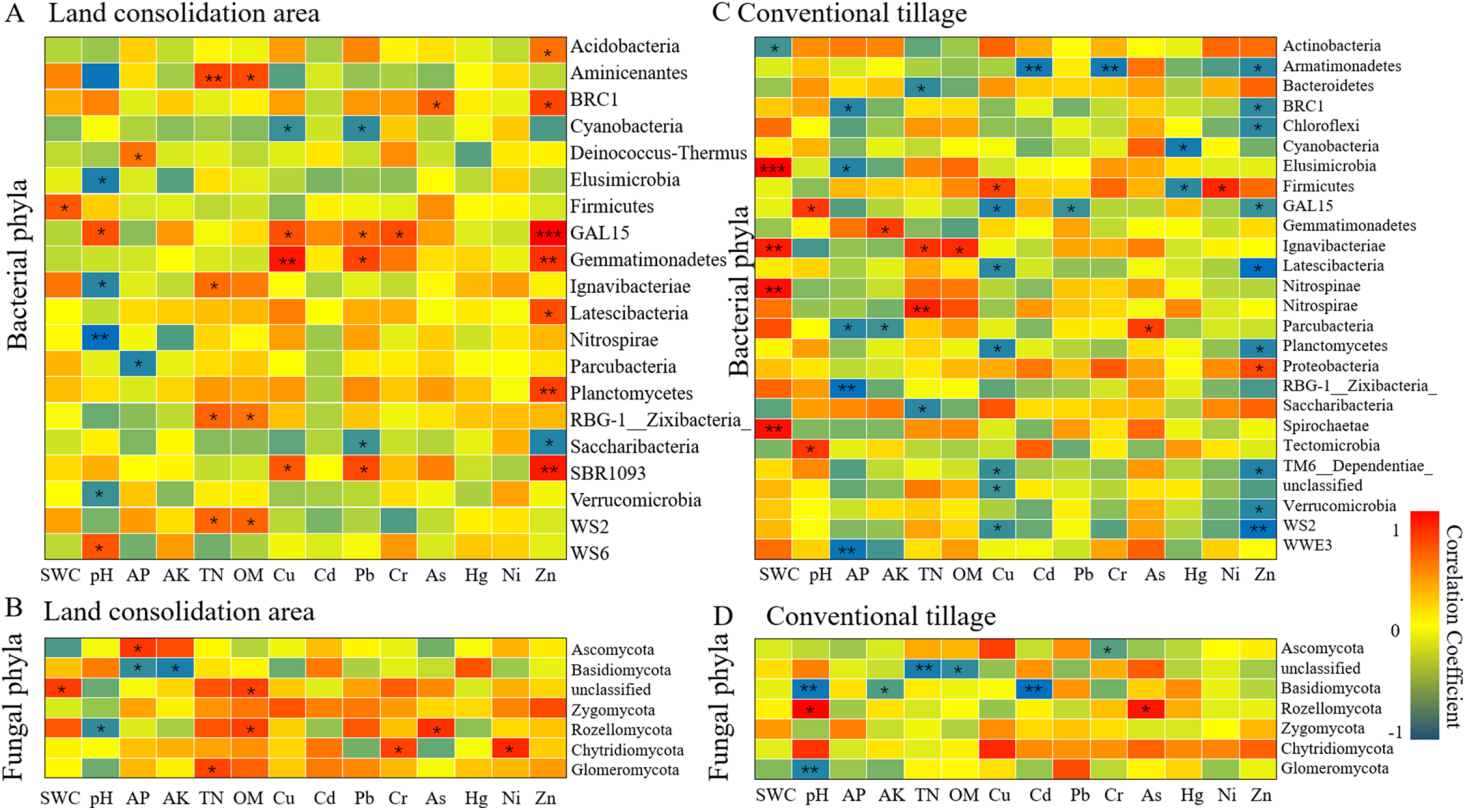
Effects of environmental factors on the community structures of dominant bacteria and fungi on phylum level by spearman analysis. The figure shows the correlation between environmental factors and microorganisms, with warm tone indicating positive correlation and cool tone indicating negative correlation. (A) Bacterial phyla in land consolidation area. (B) Fungal phyla in land consolidation area. (C) Bacterial phyla in conventional tillage area. (D) Fungal phyla in conventional tillage area. *Indicate 0.01 < *p* ≤ 0.05, **indicate 0.001 < *p* ≤ 0.01, ***indicate *p* ≤ 0.001.

At present, plenty of researches have confirmed that some microorganisms have strong function of heavy metal degradation (Xu et al., 2019; Zhao et al., 2019), and our results showed that the heavy metal tolerant species can be selected by land consolidation under the environment polluted by heavy metals. The spearman analysis (Fig. 8) illustrated that a large number of dominant bacteria are in a significant positive correlation with heavy metals of Zn, Pb and Cu (*p* < 0.05), including *Gemmatimonadetes*, *Latescibacteria* and *Planctomycetes* in the land consolidation area, which was consistent with the previous researches (Lin & Pan, 2015; Li et al., 2017). However, more dominant bacteria in the CL area were in a significant negative correlation with the concentrations of heavy metals compared with other areas. According to the spearman analysis, the dominant fungi of *Chytridiomycota*, *Glomeromycota*, *Zygomycota* and *Rozellomycota* showed a stronger correlation with heavy metal contamination and the nutrients of OM and TN in the land consolidation area than the CL area. It showed that many fungi can form a strong heavy metal adsorption function under the supplement of nutrients, and the land consolidation could create appropriate environment (Sánchez-Castro et al., 2017). Such result confirmed that land consolidation contributed to the abundance of heavy metal tolerant species.

### Soil bio-indicators under different land consolidation practices

The soil bio-indicators of microbial biomass, microbial diversity and BFD were significantly improved under land consolidation. The soil environment plays a decisive role in the suitability and availability of microorganism habitats (Murugan et al., 2019). The previous researches have demonstrated that microbial biomass accumulation is related to microbial growth in a suitable soil environment (Li et al., 2018a; Wang et al., 2019). It provides a theoretical reference to explain the various distributions of bacterial biomass, microbial diversity and BFD in these land consolidation practices. Similarly, we found that the higher values of microbial biomass, microbial diversity and BFD were mainly concentrated in land consolidation areas, particularly soils applied with organic fertilizer. Theoretically, the higher the microbial biomass and diversity are, the higher the soil quality will be.

Furthermore, the relative abundance of beneficial bacteria like *Rhizobium* and beneficial fungi like *Glomerales* and *Hypocreales* in the soils from land consolidation regions were significantly higher than others. According to the previous researches, these beneficial soil microbes can accelerate the circulation of nutrients and degradation of the heavy metals in the soil (Antoun, 2012; Chen et al., 2012; Hayat et al., 2010). This study confirmed that land consolidation was helpful to improving the relative abundance of beneficial microbes, including the species with heavy metal tolerance. These results can provide reference for the research and development of bioremediation technology in land consolidation.

### Soil quality under different land consolidation practices

Land consolidation is commonly applied to paddy fields as a major measure to improve the physicochemical and biological properties of the soil. However, it is still unknown which land consolidation measures are most effective in improving soil quality (Wang et al., 2018b). Nonetheless, our research provides evidence that all land consolidation practices can alter the soil physicochemical and biological properties to improve soil quality in paddy fields.

Based on the soil quality evaluation system proposed by the previous scholars, we believe that it is reasonable to combine the heavy metal pollution, soil physiochemical properties and biological indicators to get comprehensive results on PSQ. According to the linear fitting carried out in Fig. 7A, the values of PA exhibited an obvious increasing trend when the all soil samples were ranked in decreasing PSQ values. Comparatively, the PB values showed a slight decreasing trend. The soils under land consolidation exhibited higher values of PB and lower values of PA, so the soil quality would be relatively higher. Due to the application of chemical fertilizers, industrial wastewater discharge and atmospheric deposition may be the main sources of heavy metal pollutant in paddy fields (Deng et al., 2019). The content of pollutants can be effectively reduced through land consolidation. AO, DC, LM and LL can effectively increase the circulation of soil moisture and nutrients, promote the growth of beneficial microorganisms, thereby transforming heavy metals and improving the soil quality. This is consistent with the experimental results.

## Conclusion

This study showed that paddy soils were contaminated by heavy metals in different degrees, especially by Cd. However, the soil pH and heavy metal concentrations decreased after land consolidation, and the other physiochemical properties like OM, SWC, TN, AP and AK were increased in the soils under land consolidation. Meanwhile, the microbial biomass, microbial diversity and BFD were developed under land consolidation, particularly under the AO. The results also showed the relative abundance of beneficial bacteria like Bacillus, Bradyrhizobium and beneficial fungi like Glomerales and Hypocreales in the soils under land consolidation were much higher than others. The PSQ was positively correlated with land consolidation, and the results exhibited that all four practices can improve the PSQ to reach a satisfactory state with relatively low pollution levels. This paper innovated the comprehensive assessment of soil quality based on biological indexes, and the results also confirmed the importance of microorganisms in soil pollution remediation and nutrient cycling, which could help us better understand soil quality.

## Supplemental Information

10.7717/peerj.7351/supp-1 Supplemental Information 1 Relevant indicator data. Data of soil physical and chemical properties, heavy metal content and microbial indicators of all samples in this work.

## Additional Information and Declarations

### Field Study Permissions

The following information was supplied relating to field study approvals (i.e., approving body and any reference numbers): Field experiments were approved by the from Land Academy for National Development (LAND) of Zhejiang University to Cifang Wu.

### Data Availability

The following information was supplied regarding data availability: Data is available at NCBI SRA: PRJNA532470 and PRJNA532482 and Figshare: Lin (2019): soil quality. figshare. Dataset. https://doi.org/10.6084/m9.figshare.7991315.v1.
